# Lights Out! Nano-Scale Topography Imaging of Sample Surface in Opaque Liquid Environments with Coated Active Cantilever Probes

**DOI:** 10.3390/nano9071013

**Published:** 2019-07-14

**Authors:** Fangzhou Xia, Chen Yang, Yi Wang, Kamal Youcef-Toumi, Christoph Reuter, Tzvetan Ivanov, Mathias Holz, Ivo W. Rangelow

**Affiliations:** 1 Department of Mechanical Engineering, Massachusetts Institute of Technology, 77 Massachusetts Avenue, Cambridge, MA 02139, USA; 2 Nano analytik GmbH, Ehrenbergstraße 1, 98693 Ilmenau, Germany

**Keywords:** atomic force microscope, active cantilever probe, coating, thermomechanical actuation, piezo-resistive sensing, nano-positioner, digital lock-in amplifier, topography imaging

## Abstract

Atomic force microscopy is a powerful topography imaging method used widely in nanoscale metrology and manipulation. A conventional Atomic Force Microscope (AFM) utilizes an optical lever system typically composed of a laser source, lenses and a four quadrant photodetector to amplify and measure the deflection of the cantilever probe. This optical method for deflection sensing limits the capability of AFM to obtaining images in transparent environments only. In addition, tapping mode imaging in liquid environments with transparent sample chamber can be difficult for laser-probe alignment due to multiple different refraction indices of materials. Spurious structure resonance can be excited from piezo actuator excitation. Photothermal actuation resolves the resonance confusion but makes optical setup more complicated. In this paper, we present the design and fabrication method of coated active scanning probes with piezoresistive deflection sensing, thermomechanical actuation and thin photoresist polymer surface coating. The newly developed probes are capable of conducting topography imaging in opaque liquids without the need of an optical system. The selected coating can withstand harsh chemical environments with high acidity (e.g., 35% sulfuric acid). The probes are operated in various opaque liquid environments with a custom designed AFM system to demonstrate the imaging performance. The development of coated active probes opens up possibilities for observing samples in their native environments.

## 1. Introduction

An Atomic Force Microscope (AFM) is a nano-scale topography imaging tool widely used in high-tech industries and nanotechnology researches [1]. AFMs can conduct imaging in ambient air, transparent liquids and ultra-high vacuum (UHV) without special requirements on the sample (e.g., conductivity). The ability to take images in diverse environments makes the AFM an ideal tool for chemistry and biology researches where liquid and solid interfaces exist quite often. With the presence of a cantilever probe, the amplitudes, phases and frequencies of various modes of resonance can be utilized. This permits mapping of mechanical characteristics such as stiffness and damping in addition to topography. Moreover, the AFM can create nanoscale patterns with lithography techniques using a conductive probe or obtain mapping of sample chemical identity when combined with optical spectroscopy techniques.

Conventional AFMs utilize an Optical Beam Deflection (OBD) system to amplify the cantilever probe deflection caused by the nano-scale topography variation. In this setup, a beam of monochromatic coherent light, typically produced by a laser source with mode hopping compensation circuitry, is focused on the reflective back of the cantilever probe at an angle. The small deflection of the cantilever probe tip causes a small change in relative angle between the incident light and reflective probe back. The optical path amplifies this change to a larger movement and a four quadrant photodetector is used to convert it into an electrical signal for data acquisition systems to process. Successful implementation of the OBD system allows low-noise sub-nanometer resolution for topography measurement. With a typical size on the order of tens of microns, the laser spot size can be further reduced with high numerical aperture objective lens to work with smaller cantilever probes [2] in high-speed AFM imaging systems. However, the OBD system suffers from several limitations. First, the monochomantic light utilized in the OBD system requires optical transparency between the laser source and the back surface of the probe. This requirement prevents the application of conventional AFMs to environments with opaque liquids. Second, the alignment of the micron scale laser spot with the cantilever probe tip can be a time consuming task. The alignment can be even more difficult when operating in transparent liquid environments due to the multiple changes of refraction indcies for different materials. Third, in tapping mode operation, spurious modes of resonance can be excited in the structure from vibrating piezo element [3]. Photothermal actuation can alleviate this phenomenon but complicates the optical system alignment even more. Last but not least, the requirement of fixed relative position between the probe and the bulk optical components makes probe scan challenging to implement.

On the other hand, opaque liquid environment has practical importance in many areas of research. For example, in the refinery industry, crude oil is produced from a porous network of minerals (e.g., mica, silica, etc.) with mixture of oil and water. In this case, the wettability of the mineral determines the fluid distribution. This in turn affects the flow transport in crude oil reservoirs [4]. As crude oils contain many polar compounds, these compounds can be absorbed by the minerals and alter the effective wettability for oil or water. Hence, it is important to understand the residual formation of crude oil components on the minerals in the reservoirs. Apart from the conventional measurement method of wetting angle, previous AFM studies focused on nano-mechanical properties of residual formation after oil exposure [5]. Attempts to mimic the crude oil natural environment has been made with imaging in transparent liquid such as water or decane for AFM imaging [6]. However, due to the requirement of optical transparency, conventional AFM setup with OBD system cannot be utilized to image a wide variety of crude oil samples where non-hydrocarbon with dark color is present in the mixture. Another important area of application that could benefit from opaque liquid imaging capability is in biology research. In the case of the most common fluid in human body, namely, whole blood, AFM images of cells cannot be conducted directly with conventional AFM due to the opacity. For imaging, red blood cells are typically isolated from the plasma, treated with glutaraldehyde to render them rigid and attached to glass slide with coating (e.g., fibrinogen) [7]. Although the treatment procedure allows conventional AFM imaging of red blood cells, the treatment not only removes the cells from their native environment but also kills them outright by severely impacting their biological functionality. In addition, there are many other potential applications of opaque liquid AFM imaging in chemistry and material science that involve non-transparent liquid reaction on solid surfaces.

With the development of micro-machining and lithography technology for integrated circuit manufacturing, active cantilever probes with self-sensing and actuation can be fabricated. For deflection sensing, a piezo-resistive element embedded at the base of the cantilever probe can be connected with a circuit to provide a voltage signal proportional to the probe tip displacement. Actuation of the probe for dynamic mode operation can be achieved mechanically by conventional piezo actuators, photothermally by laser or thermomechanically with built-in heating elements. Active cantilever probes have demonstrated their capability for tapping mode AFM imaging. However, the functional components on the active cantilever probes can be electrically shorted or mechanically damaged by corrosive chemicals. If proper protective coating can be applied to the active probe without severely altering its functionality, coated active cantilever probes can be a promising technology to enable opaque liquid imaging for AFM systems.

In addition to imaging environment, size of the imaging area, spatial resolution and imaging speed (line rate) are all important considerations for the design of an AFM imaging system. A versatile imaging system is needed for various scenarios. Previous research efforts have focused on improving imaging speed of conventional AFM system by designing new generation scanners [8], utilizing smaller size cantilever probes with higher resonance frequencies [2], investigating new imaging modes [9,10], etc. As the imaging system throughput is proportional to the product of size, resolution and speed, simultaneously achieving high performances in all aspects is ideal but challenging [11]. Imaging priorities differ greatly between different imaging applications. For example, semiconductor inspection aims to cover large areas of a static sample surface whereas biological applications benefit from high-speed small area imaging for dynamic phenomenon observation. In many material science and chemistry experiments, a preliminary overview of the sample surface is ideal, from which areas of particular interest can be identified for high-speed scanning. In general, utilizing multi-actuation to cover various combination of scanning range and imaging speed helps to improve versatility of AFMs.

In this work, we present the design and application of coated active cantilever probes for topography imaging in non-transparent liquid environment with a custom built versatile AFM system. The paper is organized as follows. First, we introduce the design, fabrication, coating and characterization of the active cantilever probe. Second, we present the overall design and instrumentation of the custom imaging system capable of large range overview and high-speed local imaging. Third, we provide topography imaging results obtained from various opaque liquid environments with coated active cantilever probes in tapping mode. Finally, we discuss the potential improvements for the coating of active probes for better imaging performance.

## 2. Coated Active Cantilever Probe

**Figure 1:**
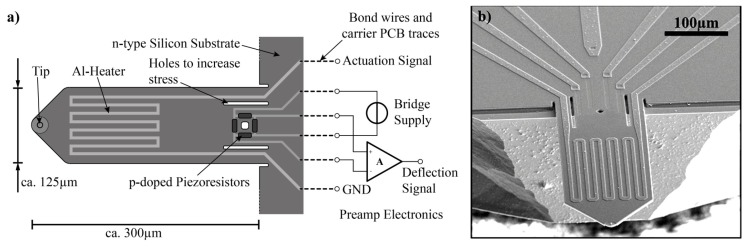
Active probe design (**a**) schematic illustration of the active probe with thermomechanical actuation and piezoresistive sensing, (**b**) SEM image of a fabricated active probe before coating.

**Figure 2:**
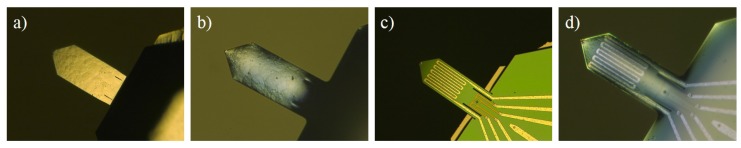
Optical microscope imaging of active probe (**a**) uncoated silicon surface, (**b**) coated silicon surface, (**c**) uncoated surface with heating wires, (**d**) coated surface with heating wires.

The key to enable opaque liquid environment imaging is a coated active probe. The design and manufacturing of an active probe with nano-fabrication technology is an intricate process that requires significant investment of time, capital and effort. At such cost, the developed active probes offer a number of benefits. First, the removal of the OBD system for deflection sensing enables easy exchange of probes without the need of laser alignment and reduces the size of the system. At such a size an AFM can even be integrated into a Scanning Electron Microscope (SEM) system [12]. Second, multiple active probes can be fabricated to form an array for simultaneous imaging of a large area [13]. Third, the ability to fabricate and embed electronics into the cantilever probe itself allows various functionalized probes to be developed as a toolkit for specific applications [14]. A more detailed discussion of the benefits, limitations and applications of active probes can be found in this review [11]. In this section, we provide an overview of the active probes for their design (Figure 1), fabrication, coating (Figure 2) and characterization of properties.

### 2.1. Design and Fabrication of Active Probes

Conventional AFM probes are etched from silicon wafers. Different choices of material, sizing and specialized probe tip treatment can be designed for different applications. In addition to the cantilever, there are two major functional subsystems embedded into our active probe, namely, (1) a piezoresistive stress sensor for probe deflection measurement and (2) thermomechanical actuator. A schematic illustration and an SEM image of the uncoated active probe is shown in Figure 1.

For cantilever deflection sensing, p-doped piezoresistors are placed at high stress locations for bending. In the case of a multi-layer cantilever, the displacement is related to the stress between layers of maximum thermal expansion coefficient difference [14]. The mechanical stress results in a specific resistance change that can be measured as a voltage signal if the piezoresistor is biased at a fixed current. By doing so, a linear relationship can be derived between the voltage across the piezoresistor and the cantilever deflection [15]. However, the presence of parasitic effects and temperature drifts can introduce noise and compromise the signal integrity especially when thermomechanical actuation is utilized. To resolve this issue, an integrated Wheatstone bridge configuration is utilized to compensate for the temperature drift and crosstalk [16,17]. Signal to noise ratio similar to optical-read-out technique can be achieved for the purpose of AFM imaging [18].

Thermomechanical actuation is achieved by resistive heating of the cantilever formed by multiple layers with different coefficients of thermal expansion. To be specific, a 6μm thick silicon layer, a 0.35μm thick ${\mathrm{SiO}}_{2}$ layer and a 0.7μm thick metal heater layer are combined. As only heating and energy dissipation into the surrounding environment is available (no cooling), unidirectional bending of the cantilever probe can be achieved. A linear relationship between temperature rise and the cantilever tip deflection can be derived [15]. For driving the probe, the situation is more complicated as the temperature is proportional to the power, which is related to the square of the supplied voltage. This non-linearity is accounted for with a custom lock-in amplifier discussed in Section 3.2.

Fabrication of the cantilever probe involves multiple lithography steps on an N-type silicon substrate. The tip of the probe is formed first and then the piezoresistor deflection sensing element with boron diffusion. After that, the heating element is formed by metal etching of an aluminum deposition layer masked with photoresist. Other types of alloys such as Cu/Au/Ta alloy with high expansion coefficient can also be utilized. A bonding process attaches the cantilever probe onto an SD card shaped PCB for electrical contact and easy probe exchange. A detailed step-by-step illustration of the cantilever fabrication process can be found in reference [15].

### 2.2. Coating of Active Probe

An uncoated active probe is suitable for operation in ambient and vacuum environments. For operation in liquid environments, it is necessary to protect the probe with coatings for electric insulation and resistance to corrosive chemicals. The coating should satisfy several requirements as discussed below to insure that it does not compromise the functionality of any subsystem of the active probe.

1. Bond well to the surface material of the cantilever probe (Silicon, ${\mathrm{SiO}}_{2}$, Heating metal, etc.)
2. Be thermally conductive with low thermal resistance to allow heat dissipation into the environment for thermomechanical actuation.
3. Form a thin uniform layer of coating without significantly affecting the mechanical properties of the probe (e.g., small additional mass, low residual stress, small increase of overall bending stiffness or damping, etc.).
4. Be chemically inactive for selected environments depending on the application (e.g., crude oil, whole blood, sulfuric acid, etc.).

Due to the delicate nature of the active cantilever probe and complication in redesigning the manufacturing process, dip coating of the active probe is selected as the fabrication method for trying out different materials for coating. In this work, various types of polymers have been evaluated including M-Bond 610, 2K.-Epoxy, M-Bond 43B and photoresist polymers for photolithography. Most of these tested materials except “Positiv 20” suffer from poor bonding, thick coating layers, inability to withstand corrosive chemicals or failure to maintain a usable resonance peak for the probe. Before the coating of the MEMS cantilever, we first protected the bond wires connecting the silicon chip with the carrier PCB. For this we used a viscous two-compound epoxy glue EPO-TEK H70E, which itself is not suitable to coat the actual cantilever beam. The epoxy was cured at 100 ${}^{\circ }$C for 2 h. The probes were then coated using the photoresist “Positiv 20” manufactured by CRC Industries Europe NV. It is primarily intended to be used as a photoresist for masking printed circuit boards. According to the datasheet, it is able to withstand strong etchants, such as hydrofluoric acid (40%), nitric acid (65%) and iron(III) chloride. Furthermore, it has a suitable viscosity to cover a silicon microstructure like an AFM cantilever with a resist layer of only a few μm thickness. Since this particular resist is sold in spray cans only, we first sprayed the aerosol into a disposable aluminum pan until a sufficient amount of resist had accumulated. Then we immersed the cantilever into the liquid, making sure that the resist covered all the parts of the silicon chip which had not been covered previously by the two-compound epoxy. Subsequently, we placed the cantilevers on a hot plate at 70 ${}^{\circ }$C for 15 min in a dark environment to dry the resist. Afterwards, we exposed the cantilevers to ultraviolet light for five minutes. Finally, we immersed the coated cantilevers into a sodium hydroxide solution (7 g/1 L) to develop the resist. Optical microscopy images as shown in Figure 2 are used as a preliminary step to verify the success of the coating application.

### 2.3. Active Probe Mechanical Property Characterization

**Figure 3:**
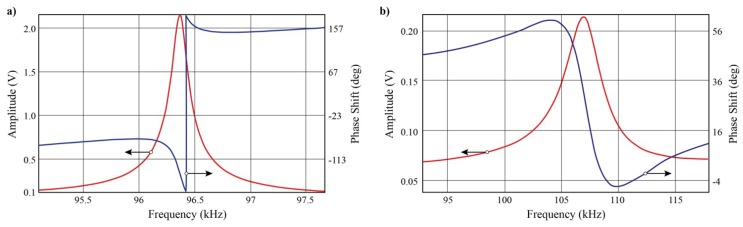
Frequency sweep results with amplitude (red), phase (blue) for one cantilever probe (**a**) before coating with resonance frequency at 96.365 kHz, (**b**) after coating with resonance frequency at 106.564 kHz (oscillation amplitudes in unit of volts are recorded instead of gains from the data acquisition system with the same driving signals applied during the sweep for comparison.).

To better understand the effect of coating, frequency response experiments were conducted for the cantilever probes before and after coating, The results are shown in Figure 3.

After the coating application, the overall mass of the probe increases, which should in principle reduce the resonance frequency of the probe. However, all probes after coating demonstrated an increase in the first mode resonance frequency. This indicates the coating increases the stiffness of the cantilever probe, which results in an overall increase of resonance frequency. The oscillation amplitude of the probe also decreased to be almost an order of magnitude smaller than the original uncoated probe with identical driving signals. This can partly be attributed to the increase of probe stiffness or damping. Another possible reason is that the coating increases the thermal capacitance and resistance of the probe such that the temperature variation amplitude is decreased. This results in a smaller probe deflection that is measured from the frequency sweep. The decrease of oscillation gain amplitude can partly be compensated by increasing the driving signal but might result in a reduced operation lifetime of the probe due to extensive heating and increased internal stress.

## 3. AFM Imaging System Design

**Figure 4:**
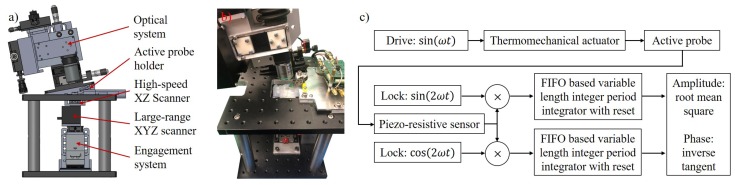
AFM system setup overview (**a**) design of the AFM with subsystem labeling in Solidworks software, (**b**) picture of the assembled AFM setup, (**c**) block diagram of the new FPGA based high throughput digital lock-in amplifier for active probe AFM operation with driving frequency ω at half of the selected operating frequency $\omega_{operate}$ slightly below the probe resonance frequency.

To evaluate the imaging performance of the coated active probes, a custom AFM system has been designed. The setup incorporates both active probe and OBD system passive probe imaging with a sample scan configuration. An overview of the AFM system Solidworks design and picture of the assembled setup is shown in Figure 4a,b. In this section, we provide a general introduction of the subsystems and elaborate on the FPGA demodulation implementation for active probe imaging.

### 3.1. System Level Design

To cater to various types of research demands, a 5-axis scanner with multi-actuation is designed. For large range overview of the sample, a P-611.XYZ from Physik Instrumente (PI) is utilized. The scanner operates in 3 orthogonal axes at 100 μm positioning range with capacitive sensor feedback for high positioning accuracy with resonance frequency up to 180 Hz. To allow high-speed scanning capability, a high-bandwidth scanner for X and Z axis operations is designed. For the high-speed Z axis, a PL022.31 piezo actuator from PI constrained by a flexure cap is used to achieve bandwidth over 100 kHz with 1 micron motion range. In the X-axis direction, 2 P-885.11 piezo actuators from PI are utilized to form a dual push configuration. The sample stage is constrained via music wires on a set of 8 ball bearings. This configuration reduces the crosstalk between axis and minimizes the thermal drift of piezo actuators at high bandwidth. The X-axis has a 10 kHz bandwidth with maximum of 6.5 micron travel range. Although not being the focus of the this preliminary study on coated active probe performance, the high-speed scanner offers potential for dynamic phenomenon visualization with more details available in reference [19]. High-speed imaging using coated active probes would require contact mode operation with a coating material that maintains sharpness of the probe tip.

The high-precision engagement system is constructed by combining a PK523HPA-H50S harmonic drive stepper motor from Oriental Motor Corporation capable of 25,000 steps per revolution with a DS40-XYZ positioner with 80 revolution per inch accuracy. The combined setup has a theoretical 12.7 nanometer step size that allows precise control of the engagement process. The active probe holder includes mounting structure and signal conditioning circuitry. The optical system utilizes infinite corrected objective lens to allow optical microscope view and optical readout for passive probes. External drivers with piezo actuator hysteresis compensation capability to improve positioning accuracy without usage of feedback sensor [20] are also developed.

### 3.2. FPGA Based High Throughput Digital Lock-in Amplifier

Due to the thermomechanical actuation of the active probes, the driving method is different from conventional tapping mode AFM imaging with passive probes. The relationship between the deflection of the probe and input voltage with DC and AC components is given in Equation (1).

(1) $$U_{p}\propto \delta \propto T\propto P_{heat}=\frac{{\left(V_{dc}+V_{ac}\sin\left(\omega t\right)\right)}^{2}}{R}=\frac{1}{R}\left(V_{dc}^{2}+2V_{dc}V_{ac}\sin\left(\omega t\right)+\frac{1}{2}V_{ac}^{2}\left(1-\cos\left(2\omega t\right)\right)\right)$$

where $U_{p}$ is the voltage signal from the piezoresistor readout, δ is the cantilever probe deflection, *T* is the temperature, $P_{heat}$ is the resistive heating power, *R* is the heater electrical resistance, $V_{dc}$ is the DC component of the applied voltage, $V_{ac}$ is the magnitude of the AC component of the applied voltage, ω is the driving frequency in rad/s and *t* is time. We implicitly utilized the trigonometry identity $2\sin^{2}\left(\omega t\right)=1-\cos\left(2\omega t\right)$ in the derivation. From Equation (1), the output signal can be considered as having 1 DC component and 2 AC components at frequencies ω and 2ω. To improve the demodulation performance, it would be beneficial to eliminate one of the AC components. A straightforward method to achieve this goal is to set $V_{dc}=0$ such that the AC output of the power is at the frequency of 2ω. This essentially means that the driving sinusoidal waveform frequency ω is at half of the selected operating frequency $\omega_{operate}$ slightly below the first resonance of the cantilever probe to have $\omega =\omega_{operate}/2$.

To improve the demodulation speed, integration over integer number of periods is preferred over long time integration with low pass filter. Based on previous research, the oscillation amplitude can be extracted with only a few periods of the sinusoidal waveform [10]. With proper implementation of a pipeline for integration, the digital lock-in amplifier can produce amplitude value at the same throughput rate as that of the data acquisition. It is also adjustable to a wide range of frequencies (mHz to MHz) with minimal added latency. Figure 4c provides a block diagram overview of the design.

For implementation, the lock-in amplifier is realized with NI LabVIEW on an PXIe-7975R FPGA board with NI-5782 high bandwidth digital IO at 125 MHz sampling rate. Special care is taken to minimize the FPGA fixed point arithmetic quantization error for precise integer periods integration. The high-speed PXIe-7975R FPGA processes data from the active probe readout circuitry and provides the lock-in amplifier output to the PXIe-7868R FPGA, which handles the scanning pattern generation, regulation and image data acquisition. An event driven state machine is implemented in real time for user interface event handling, controller tuning and FPGA resource coordination. Real time 2D/3D topography visualization and recording is also implemented for user convenience considerations.

## 4. Experimentation

In this section, we provide imaging results in various environments with the coated active probes. For consideration of signal quality and the bluntness of the active probe tip due to coating, amplitude regulation tapping mode is more suitable than contact mode for better imaging resolution. We evaluated the imaging performance of coated active probe comparatively with uncoated active probe and investigated the functionality of each in different chemical and biological environments. With tapping mode operation and cosidering feature size of the sample, we utilized the large range scanner to cover areas of 6 by 6 micron for oil imaging and 12 by 12 micron for acid imaging respectively.

### 4.1. Comparative Imaging with Crude Oil Environment

**Figure 5:**
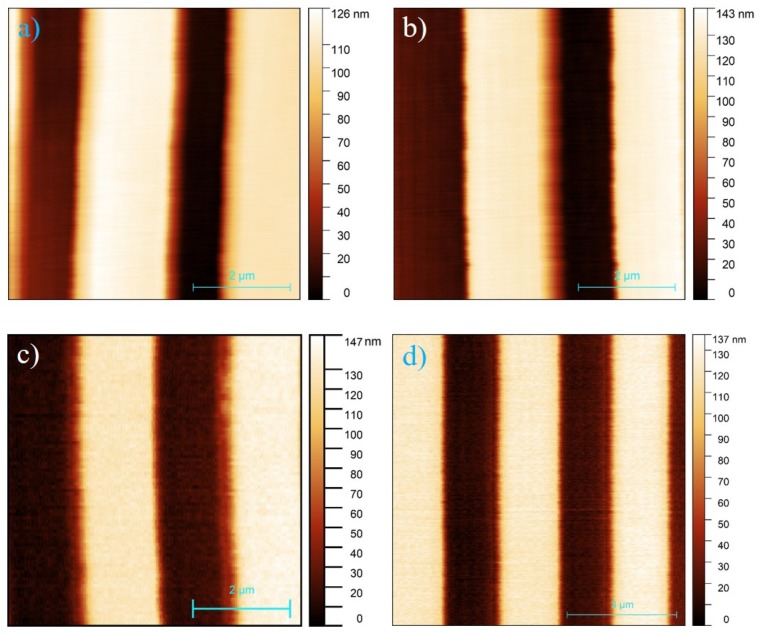
Imaging of TGZ-100 calibration sample with 6 by 6 micron size (a to c) for at 400 by 400 pixel resolution in tapping mode at 4 lines per second (**a**) uncoated regular active probe in air, (**b**) coated probe in air, (**c**) coated probe in crude oil environment, (**d**) coated probe in crude oil with 8 by 8 micron size and 600 by 600 pixel resolution image at lower speed 0.5 line per second for better edge resolution.

To evaluate the capability of the coated cantilever probe and obtain a better understanding of liquid environment imaging performance, the same sample was imaged with uncoated active probe in air, coated active probe in air and coated active probe in crude oil. The results are shown in Figure 5 for comparison. In this case, a TGZ-100 calibration grating sample from Ted Pella Inc with trenches at 110±2 nm step height and 3±0.01μm pitch period was imaged. We used a crude oil sample obtained from South Dakota, USA for the opaque liquid environment.

As can be seen in Figure 5, the height sensitivity of the coated active cantilever probe is comparable to the uncoated probe. With tapping mode imaging of step trenches, a ”parachuting effect” occurs when a sudden height reduction is scanned in the topography. The cantilever needs time to reach a larger oscillation amplitude that in turn drives the controller as it tracks the topography. Slower imaging speed, high resonance frequency and smaller oscillation amplitude setpoint helps reduce this effect but at the cost of longer imaging time and lower sensitivity. From the images (a) and (b) in Figure 5, the coated probe actually demonstrated less parachuting effect, evidenced in the more evenly spread pattern in Figure 5b. This can be attributed to the smaller oscillation amplitude and setpoint for the coated active probe since other imaging parameters and imaging environment are identical. On the other hand, the topography edge noise of the coated probe operating in oil is higher than uncoated probe in air at 4 lines per second scanning rate as can be compared between Figure 5a,c. By lowering the scanning rate, the noise can be reduced as shown in Figure 5d. Hence, the opaque liquid imaging capability of the coated active probe is demonstrated in tapping mode.

### 4.2. Chemical Inertness Verification in Corrosive Environment

**Figure 6:**
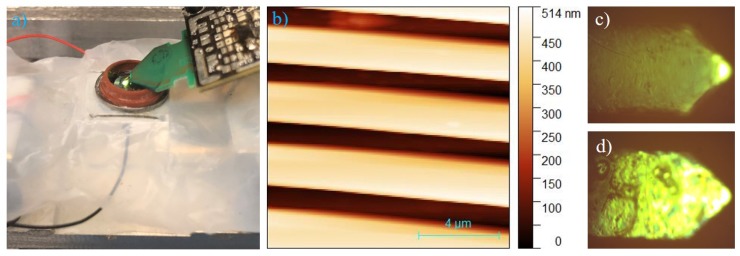
Acid imaging (**a**) sample in vinegar on the teflon sheet protected scanner, (**b**) tapping mode imaging of calibration grating with Z=520±3 nm step height and 3±0.01μm pitch period from Ted Pella Inc with 12 micron by 12 micron range at 2 lines per second, (**c**) microscope image of coated active probe before experiment and (**d**) microscope image of probe after long time operation demonstrated partial coating detachment (probe is still functional and coating does not react with vinegar).

To investigate the capability of the coated probes to resist corrosive environments, acid environment imaging was conducted with a larger sample height variation. In this case, vinegar with black color with pH value around 3 was used. Sample setup of a TGZ-500 calibration grating, imaging result and visualization of probe coating changes after imaging are shown in Figure 6.

The coated active probe performs well compared to the uncoated probe despite its relatively smaller oscillation amplitude and sample’s large step height variation. Despite the visible change of the coated probe back surface as shown in Figure 6c,d due to bonding detachment, the active probe continues to function well in the acid imaging environment for extended time over several hours. This implies that the coating effectively insulate the probe from electrical and chemical reaction with the liquid environment. Further discussion on this observation is provided in Section 5.2.

### 4.3. Biological Sample Immersion Test

Immersion test for whole blood sample with Ethylenediaminetetraacetic acid (EDTA) anti-coagulation treatment from AllCells LLC was also conducted. The coated probe worked well at the beginning of the immersion in terms of resonance identification. However, due to the difference in densities of its components, the blood sample formed a deposition of multiple layers. In addition, the presence of heterogeneous floating cells affected the resonance frequency of the probe. These complications made the probe engagement with the sample surface a time consuming task. Furthermore, even with anti-coagulation treatment, the blood sample solidified within approximately 30 min of exposure to ambient air by forming an outer shell at the air-blood interface. This phenomenon significantly affects the resonance of the probe and can even break the active probe. Hence, the coated probe is functional in a whole blood sample but effective imaging will require specialized treatment with chemical agents or dedicated environmental control to prevent blood sample solidification.

## 5. Discussion

A number of observations were made during the imaging process that are each worth further discussion. In this section, we share our experiences with the AFM imaging of opaque liquids using coated active probes. The report will also include observations of probe coatings after extended time of usage. Potential improvements of the coating for better imaging performance are also discussed.

### 5.1. Liquid Operation Considerations

A number of issues can arise when operating in liquid environments. One of these is that upon moving the probe from a gas (air) into a liquid environment, its resonance frequency often shifts due to added mass of the liquid. This shift can be confused for engagement with the sample for reduced oscillation amplitude. Several other resonance frequency shifts occur at the end of the engagement upon approaching the solid surface. Hence, several cantilever frequency sweeps need to be conducted to ensure the actual surface of the sample is reached.

Second, depending on the density and viscosity of the liquid, the first resonance frequency can become significantly lower such that the amplitude demodulation speed is too slow. In this case, second mode of the resonance frequency can be utilized for imaging.

Finally, due to the surface tension and wetting effects of liquids, residuals of the liquid remain on the polymer coating of the probe after experiment. After drying in air, the residual particles can change the mechanical properties of the probe or react with liquid in the next experiment. Therefore, it is necessary to clean the probe in chemical solution to dissolve residual particles. The probe needs to be rinsed in deionized water and then dried in a clean environment to avoid dust attachment.

### 5.2. Mechanical Effects of Coating

In addition to the increase of probe mass, stiffness and resonance frequency as demonstrated in Section 2.3, the coating creates an additional non-linear effect after extended time of operation. From optical microscope videos of actuation of a coated active probe in air, visual changes of the coating can be observed as shown in Figure 6c,d. As the chemical reaction inertness of the coating is verified during material selection stage as described in Section 2.2, the phenomenon was potentially due to the repeated oscillation and thermal heating of the probe causing the coating to partially detach from the probe surface. Although the coating protection against liquid was still functional, the probe started to exhibit non-linearity. The resonance frequency started to depend on the driving amplitude and distortion could be observed in the probe oscillation waveform. This phenomenon is less than ideal as it increases the complexity of the imaging process where probe sweeping needs to be conducted for actuation at different amplitudes. Nevertheless, the imaging capability of the coated probe in harsh opaque liquid environment is enabled.

### 5.3. Potential Improvements of the Coating

By applying protective coating to the active cantilever probe, the new capability of harsh opaque liquid imaging for AFM system is enabled. As demonstrated by the imaging performance, the side effects of reduced probe sharpness and non-linearity due to thermomechanical stress induced fatigue did not reduce the usefulness of such technique for tapping mode imaging. We now discuss possible improvements and potential methods. First, the thickness of the coating should be reduced for smaller added mass. Second, the overall probe stiffness increase caused by the coating should be minimized to maintain original probe oscillation amplitude for imaging sensitivity considerations. Third, bonding strength of the coating with the probe should be improved to sustain long time operation without added non-linearity. Last but not least, increasing the overall coated area to cover the entire electronics area to allow deeper immersion into the liquid environment would be helpful for experiment purpose.

Vapor deposition techniques would be an ideal future method of adding the coating because it offers good bonding and atomic level thickness control. However, the development of such coating requires augmentation of the nano-fabrication process. This will require significant investment of time and capital for development. Another promising potential method is to apply parylene coating, which allows control of coating thickness below one micron. Parylene coating can also resist harsh chemical environments and be applied after probe fabrication with current design.

## 6. Conclusions and Future Work

In this work, we extended the capability of AFM systems to operate in opaque liquid environments. The design, fabrication and coating of active cantilever probes are presented. A versatile AFM system has been custom designed for operation in various range, bandwidth and probe configurations. Coating and system performance are verified with imaging in vinegar, crude oil, and immersion test in blood sample. Experiment key points and potential improvements are further discussed for usage of the cantilever probe. For future work, the authors will continue experimenting on other coating methods to improve the performance of coated active probes for AFM imaging in chemically harsh opaque environments.

## 7. Patents

A provisional patent has been filed for the results of this research.

## Abbreviations

The following abbreviations are used in this manuscript:

AFM	Atomic Force Microscope
EDTA	Ethylenediaminetetraacetic acid
FPGA	Filed Programmable Gate Array
MEMS	Micro-Electro-Mechanical Systems
OBD	Optical Bean Deflection
PCB	Printed Circuit Board
SEM	Scanning Electron Microscope
UHV	Ultra-High vacuum

## References

[1] Binnig G., Quate C.F., Gerber C. (1986). Atomic Force Microscope. Phys. Rev. Lett..

[2] Richter C., Weinzierl P., Engl W., Penzkofer C., Irmer B., Sulzbach T. (2012). Cantilever probes for high speed AFM. Microsyst. Technol..

[3] Asakawa H., Fukuma T. (2009). Spurious-free cantilever excitation in liquid by piezoactuator with flexure drive mechanism. Rev. Sci. Instrum..

[4] Kumar K., Dao E., Mohanty K.K. (2005). AFM study of mineral wettability with reservoir oils. J. Colloid Interface Sci..

[5] Kaimaki D.M., Smith B.E., Durkan C. (2018). On the use of nanomechanical atomic force microscopy to characterise oil-exposed surfaces. RSC Adv..

[6] Lord D., Buckley J. (2002). An AFM study of the morphological features that affect wetting at crude oil-water-mica interfaces. Colloids Surfaces A: Physicochem. Eng. Asp..

[7] Lavalle P., Stoltz J.F., Senger B., Voegel J.C., Schaaf P. (1996). Red blood cell adhesion on a solid/liquid interface. Proc. Natl. Acad. Sci. USA.

[8] Yong Y.K., Moheimani S.O.R., Kenton B.J., Leang K.K. (2012). Invited Review Article: High-speed flexure-guided nanopositioning: Mechanical design and control issues. Rev. Sci. Instrum..

[9] Miyata K., Tracey J., Miyazawa K., Haapasilta V., Spijker P., Kawagoe Y., Foster A.S., Tsukamoto K., Fukuma T. (2017). Dissolution Processes at Step Edges of Calcite in Water Investigated by High-Speed Frequency Modulation Atomic Force Microscopy and Simulation. Nano Lett..

[10] Xia F., Bozchalooi I.S., Youcef-Toumi K. Induced vibration contact detection for minimizing cantilever tip-sample interaction forces in jumping mode atomic force microscopy. Proceedings of the 2017 American Control Conference (ACC), ACC’17.

[11] Rangelow I.W., Ivanov T., Ahmad A., Kaestner M., Lenk C., Bozchalooi I.S., Xia F., Youcef-Toumi K., Holz M., Reum A. (2017). Review Article: Active scanning probes: A versatile toolkit for fast imaging and emerging nanofabrication. J. Vac. Sci. Technol. B.

[12] Rangelow I.W., Kaestner M., Ivanov T., Ahmad A., Lenk S., Lenk C., Guliyev E., Reum A., Hofmann M., Reuter C. (2018). Atomic force microscope integrated with a scanning electron microscope for correlative nanofabrication and microscopy. J. Vac. Sci. Technol. B.

[13] Ahmad A., Nikolov N., Angelov T., Ivanov T., Reum A., Atanasov I., Guliyev E., Ishchuk V., Kaestner M., Krivoshapkina Y. (2016). Large area fast-AFM scanning with active “Quattro” cantilever arrays. J. Vac. Sci. Technol. B.

[14] Abedinov N., Grabiec P., Gotszalk T., Ivanov T., Voigt J., Rangelow I.W. (2001). Micromachined piezoresistive cantilever array with integrated resistive microheater for calorimetry and mass detection. J. Vac. Sci. Technol. A.

[15] Ivanov T., Gotszalk T., Grabiec P., Tomerov E., Rangelow I.W. (2003). Thermally driven micromechanical beam with piezoresistive deflection readout. Microelectron. Eng..

[16] Rangelow I.W., Skocki S., Dumania P. (1994). Plasma etching for micromechanical sensor applications. Microelectron. Eng..

[17] Rangelow I.W., Ivanov T., Volland B., Gotszalk T., Woszczyna M., Mielczarski J., Sarov Y. (2014). Apparatus and Method for Investigating Surface Properties of Different Materials. U.S. Patent.

[18] Pedrak R., Ivanov T., Ivanova K., Gotszalk T., Abedinov N., Rangelow I.W., Edinger K., Tomerov E., Schenkel T., Hudek P. (2003). Micromachined atomic force microscopy sensor with integrated piezoresistive sensor and thermal bimorph actuator for high-speed tapping-mode atomic force microscopy phase-imaging in higher eigenmodes. J. Vac. Sci. Technol. B.

[19] Xia F., Truncale S., Wang Y., Youcef-Toumi K. Design and Control of a Multi-actuated High-bandwidth and Large-range Scanner for Atomic Force Microscopy. Proceedings of the 2018 Annual American Control Conference (ACC).

[20] Yang C., Li C., Xia F., Zhu Y., Zhao J., Youcef-Toumi K. (2019). Charge Controller With Decoupled and Self-Compensating Configurations for Linear Operation of Piezoelectric Actuators in a Wide Bandwidth. IEEE Trans. Ind. Electron..

